# Sustainability of Community-Based Specialized Mental Health Services in Five European Countries: Protocol for Five Randomized Controlled Trial–Based Health-Economic Evaluations Embedded in the RECOVER-E Program

**DOI:** 10.2196/17454

**Published:** 2020-06-01

**Authors:** Ben F M Wijnen, Filip Smit, Ana Ivičević Uhernik, Ana Istvanovic, Jovo Dedovic, Roumyana Dinolova, Raluca Nica, Robert Velickovski, Michel Wensing, Ionela Petrea, Laura Shields-Zeeman

**Affiliations:** 1 Center for Economic Evaluation and Machine Learning Trimbos Institute Utrecht Netherlands; 2 Department of Clinical Epidemiology and Medical Technology Assessment Maastricht University Medical Center+ Maastricht Netherlands; 3 Department of Biostatistics and Epidemiology and Department of Clinical, Neuro and Developmental Psychology Amsterdam Public Health Research Institute VU University Medical Center Amsterdam Netherlands; 4 Division of Public Health Croatian Institute of Public Health Zagreb Croatia; 5 Special Psychiatric Hospital Dobrota Kotor; 6 National Centre of Public Health Protection–Mental Health Sofia Bulgaria; 7 Romanian League for Mental Health Bucharest Romania; 8 University Clinic of Psychiatry Skopje the Former Yugoslav Republic of Macedonia; 9 Department of General Practice and Health Service Research University Hospital Heidelberg Heidelberg Germany; 10 Department of Public Mental Health Trimbos Institute Utrecht Netherlands

**Keywords:** community-based mental health care, cost-effectiveness analysis, cost-utility analysis, economic evaluation, mental health

## Abstract

**Background:**

Community-based recovery-oriented mental health services for people with severe mental disorders have not been fully implemented in Bulgaria, Croatia, Macedonia, Montenegro, and Romania. The RECOVER-E project facilitates the implementation of specialized mental health care delivered by setting up services, implementing the services, and evaluating multidisciplinary community mental health teams. The outcomes of the RECOVER-E project are assessed in a trial-based outcome evaluation in each of the participating countries with a health-economic evaluation linked to these trials.

**Objective:**

The aim of this protocol paper is to describe the methodology that will be used for the health-economic evaluation alongside the trials.

**Methods:**

Implementation sites have been selected in each of the five countries where hospital-based mental health services are available (care as usual [CAU]) for patients with severe mental disorders (severe depression, bipolar disorder, schizophrenia, and other psychotic disorders). The newly implemented health care system will involve community-based recovery-oriented mental health care (CMHC). At each site, 180 consenting patients will be randomized to either CAU or CMHC. Patient-level outcomes are personal and social functioning and quality-adjusted life years (QALYs). Data on participants’ health care use will be collected and corresponding health care costs will be computed. This enables evaluation of health care costs of CMHC as compared with CAU, and these costs can be related to patient-level outcomes (functioning and QALY gains) in health-economic evaluation.

**Results:**

Data collection was started in December 2018 (Croatia), February 2019 (Montenegro), April 2019 (Romania), June 2019 (North Macedonia), and October 2019 (Bulgaria). The findings of the outcome evaluations will be reported for each of the five countries separately, and the five trials will be pooled for multilevel analysis on a combined dataset.

**Conclusions:**

The results of the health-economic evaluation of the RECOVER-E project will contribute to the growing evidence base on the health and economic benefits of recovery-oriented and community-based service models for health systems in transition.

**Trial Registration:**

(1) ClinicalTrials.gov NCT03922425 (Bulgaria); https://clinicaltrials.gov/ct2/show/NCT03922425 (2) ClinicalTrials.gov NCT03862209 (Croatia); https://clinicaltrials.gov/ct2/show/NCT03862209 (3) ClinicalTrials.gov NCT03892473 (Macedonia); https://clinicaltrials.gov/ct2/show/NCT03892473 (4) ClinicalTrials.gov NCT03837340 (Montenegro); https://clinicaltrials.gov/ct2/show/NCT03837340 (5) ClinicalTrials.gov NCT03884933 (Romania); https://clinicaltrials.gov/ct2/show/NCT03884933

**International Registered Report Identifier (IRRID):**

DERR1-10.2196/17454

## Introduction

Many European countries have undergone the process of deinstitutionalization of their mental health care services. Often, this entailed a shift away from hospital-based care toward assertive community treatment (ACT) or ACT-like services for patients, such as flexible assertive community treatment (FACT). Under FACT models, patients live in the community and receive care from community mental health care teams (CMHTs) [1,2]. Typically, these teams consist of psychiatrists, psychologists, social workers, psychiatric nurses, and peers (people with lived experiences of severe mental disorders). The CMHTs focus on evidence-based (ie, guideline concordant) and patient-centered care in those domains where the patient needs recovery the most. This could be treatment directed at symptomatic remission, but could also, and just as importantly, entail support for the patient’s personal and social role functioning (eg, independent living, getting along with others, and participating in the community). In brief, the care offered by CMHTs can be described as community-based recovery-oriented models of care.

Community-based recovery-oriented mental health services for people with severe mental disorders are in the early stages of development in Bulgaria, Croatia, Macedonia, Montenegro, and Romania. The RECOVER-E project facilitates the implementation of specialized mental health care delivered by setting up services, implementing the services, and evaluating multidisciplinary CMHTs [3]. This implementation process is flanked by research from start to end. The outcomes of the RECOVER-E project are assessed in a trial-based *outcome evaluation* in each of the participating countries or sites (note that CMHTs will be used at the specified sites and not at the country level). An aspect of this outcome evaluation is the implementation of five health-economic evaluations designed alongside hybrid effectiveness-implementation trials. Finally, this outcome evaluation will result in a series of policy briefs to inform all stakeholders of the *policy dialogues* directed at national scale up of the newly implemented mental health care model and its sustainability.

The aim of this protocol paper is to describe the overall methodology of the five health-economic evaluations that will assess the incremental cost-effectiveness of CMHTs focusing on recovery-oriented care compared with care as usual (CAU). The economic evaluation will be conducted as both a cost-effectiveness analysis (CEA), where treatment response (defined as improvement in global functioning) is the primary outcome, and a cost-utility analysis (CUA) with quality-adjusted life years (QALYs) gained as the main outcome.

## Methods

### Design

The study is conducted at five sites in the cities of five countries in Central and Eastern Europe (Sofia, Bulgaria; Zagreb, Croatia; Skopje, Macedonia; Kotor, Montenegro; and Siret, Suceava County, Romania). In each country, the study is designed as a health-economic evaluation alongside a pragmatic randomized trial in two parallel groups, comparing newly implemented community-based recovery-oriented mental health care (CMHC) with CAU. Measurements will be performed at baseline (*t_0_*) and at 12 and 18 months after baseline (*t_1_* and *t_2_*, respectively). A Consolidated Standards of Reporting Trials (CONSORT) flow diagram of the study is shown in Multimedia Appendix 1.

### Selected Study Sites

In each of the five countries, one implementation site is selected, using the following criteria: demonstrated a need from stakeholders to scale up community care for people with mental ill health through policy documents, political decisions, or statements made via EU platforms such as the Joint Action for Mental Health and Wellbeing (2012-2015); firm local leadership and support for implementation from local decision-makers; and a selection of sites that reflect the diversity of health systems in Europe, the different stages of transition within the deinstitutionalization process for mental health care, and the different human and technical resources available to start implementation of a community-based mental health project. The selected sites are Mental Health Centre Prof. N. Shipkoveski Ltd. (Sofia, Bulgaria), University Hospital Centre Zagreb (Zagreb, Croatia), University Clinic of Psychiatry (Skopje, Macedonia), Psychiatric Hospital Dobrota (Kotor, Montenegro), and Siret Psychiatric Hospital (Suceava, Romania).

The trials will not start at the same time across all implementation sites in order to avoid allocating resources to all five trials simultaneously. Instead, a pragmatic approach will be adopted by starting three of the trials at three implementation sites in year 1 of the project (Croatia, Montenegro, and Romania), based on the preparedness to start the trial and the readiness of local authorities and health care professionals to start the implementation. The remaining two sites in Bulgaria and Macedonia will start the trial in year 2.

### Eligible Participants

The study participants are consenting adults (aged 18-65 years) with severe mental illness defined as follows: (1) Patients making their first entry into the mental health care system (ie, first admissions without a prior treatment history) with a diagnosis of bipolar disorder, severe major depression, schizophrenia, schizophreniform, and schizoaffective disorder according to the International Statistical Classification of Diseases and Related Health Problems-10 (not in symptomatic remission and in need for continued care) and having severe limitations in personal and social role functioning according to the International Classification of Functioning, Disability and Health (not in functional remission and in need of coordinated care provided by community mental health teams); (2) Readmitted patients who have a treatment history but make a re-entry into the mental health care system (ie, readmissions; patients who make a fresh start with treatment for a new episode or recurrence of their disorder) on meeting the diagnostic criteria for the above-mentioned diagnoses.

Patients will be excluded from participation on presenting with somatic comorbidities (ie, dementia or other severe organic causes of brain damage that can decrease their capacity to consent and participate in the study) that require prolonged medical care in a hospital, undergoing incarceration, or presenting with a terminally ill condition, which makes it impossible to either randomize them to community-based care or precludes long-term follow-up assessments in the context of the study. Patients will preferably be excluded from participation when they have a prior treatment history longer than the past 12 months (ie, from the time of possible inclusion or visit to the participating centers), because a longstanding treatment history may confound or bias the evaluation of patient-level health outcomes.

### Recruitment

Patients will be recruited from the population being treated by specialized inpatient and outpatient mental health services participating in the study. Eligible patients expressing interest to participate will receive an introductory letter, a patient information leaflet explaining the study’s aims and procedures, and an informed consent form. Patients willing to participate in the study will be asked to sign and return the informed consent form. Patients who decline participation in the study will receive CAU. Each included patient receives a unique identification number for data collection and monitoring of patient flow into and through the trial. At each site, a minimum of 180 patients and a maximum of 200 patients need to be recruited.

In line with ethical requirements, any study participant can decide to withdraw from the study at any time. In addition, responsible clinicians can decide for individual patients to deviate (temporarily or permanently) from the intervention program. Their professional autonomy and responsibility remain. Nonetheless, data analysis will be conducted in agreement with the intention-to-treat (ITT) principle, where all randomized participants are analyzed in the condition to which they were randomized.

### Intervention Condition

The CMHTs provide mental health care within a locally adapted version of FACT (thereby still allowing for flexibility due to differences in team compositions and other site-specific practices), an evidence-based service delivery model for providing services to people with severe mental illness, to attain their recovery goals, as well as timely and appropriate psychiatric care in the event of a crisis. FACT provides flexible and intensive home-based treatment to people with severe mental illness and is an adapted form of ACT; the latter approach has been widely implemented in North America, Australia, and Europe [4]. ACT is particularly effective (both clinically and cost-wise) when targeted to high users of inpatient care and has been found to be acceptable in patients [4].

### Comparator or Control Condition

Patients randomized to the control condition will be provided CAU by their respective health care organizations and accompanied providers . The constituent of usual care across the implementation sites differs, but it is mostly offered as hospital-based outpatient care (delivered within the psychiatric hospital) and inpatient psychiatric care. None of the current implementation sites have well-functioning CMHTs that provide home treatment or crisis care in the community.

### Randomization and Masking

Eligible and consenting patients will be randomly allocated to either the intervention (receiving care provided by CMHTs) or usual care condition consisting of inpatient or outpatient mental health care (described above). An independent statistician, otherwise not involved in the trial, will carry out the randomization at each of the sites, with patients as the unit of randomization. Simple randomization with 1:1 allocation will be applied using a randomization website [5] for true random number generation. In this type of study (a hybrid implementation-effectiveness trial), it is not possible to conceal the randomization status from either clinicians or patients, and masking will therefore not be attempted.

### Measures

The primary outcome is disability in personal and social functioning (henceforth referred to as *functioning*). Functioning is measured using the World Health Organization Disability Assessment Schedule 2.0 (WHODAS 2.0), which is directly linked to the International Classification of Functioning, Disability and Health [6,7]. According to the World Health Organization (WHO), the concept of disability refers to a degree of functional impairment at the bodily, social, and environmental levels, affecting everyday activities and social participation. WHODAS produces standardized disability levels, which are applicable across all diseases, including mental, behavioral, and neurological disorders, in both clinical and general population settings and across cultures. It captures a person’s functioning in the following six life domains: (1) *cognition*, understanding and communicating; (2) *mobility*, moving and getting around; (3) *self-care*, attending to one’s hygiene, dressing, eating, and staying alone; (4) *getting along*, interacting with other people; (5) *life activities*, domestic responsibilities, leisure, work, and school; and (6) *participation*, joining in community activities and participating in society. The full version of the WHODAS has 36 items. Each item is scored on a 5-point Likert disability scale (0, none; 1, mild; 2, moderate; 3, severe; and 4, extreme). The scores can be summed into an overall functional disability score or presented as six domain-specific sum scores. Alternatively, a more complex scoring algorithm (based on item-response theory) is used (available for SPSS [IBM Corp, Armonk, New York, USA]), which provides an overall score for global functioning on a scale between 0 and 100. In this study, the 36-item self-reported version will be used as a self-administered questionnaire, but trained and supervised interviewers will be available to assist the patients filling in the WHODAS if required. The WHODAS has good psychometric properties and has been designed to monitor the impacts of health and health-related interventions, with a Cronbach α of .86 (range .82-.98) [7].

The secondary patient-level outcome is health-related quality of life, which will be measured using the three-level EuroQoL five dimensions (EQ-5D-3L) [8], which is more commonly used in Eastern Europe. Moreover, a translated version of the newer five-level scale is not available for all countries at the start of the study. The EQ-5D-3L contains the following five dimensions of health-related quality of life: mobility, self-care, daily activities, pain or discomfort, and depression or anxiety. Each dimension can be rated at three levels (from no problems to major problems). The five dimensions can be summed into a descriptive health state with “11111” indicating no problems in any of the five health dimensions and “33333” indicating major problems in all dimensions. In this way, the EQ-5D-3L can describe 243 (3^5^) health states. For each of the health states, utility values can be calculated using health state preferences elicited from the Slovenian population [9], as no health state preferences are currently available for any of the five countries included in this study, and out of the available countries, Slovenia is chosen owing to its geographical proximity, as well as similar historical and social environment. For the purpose of sensitivity analyses, utility values will also be elicited using the EuroQoL visual analog scale (VAS). The utility values give weight to the amount of time that a person spends in a certain health state, which is used to compute QALYs. In the CUA, the QALYs will be the outcome of interest.

Data on *resource use* (healthcare uptake, including informal care, travel distances to health services, and productivity losses stemming from functional impairment) will be collected using an adapted version of the Trimbos/iMTA Questionnaire on Costs associated with Psychiatric illness (TiC-P) [10]. We will consider the following three types of costs: (1) health care costs; (2) out-of-pocket costs (from patients and their family members for travel and informal care); and (3) costs stemming from productivity losses due to absenteeism and lesser efficiency while at work (presenteeism). Costs will be estimated using a bottom-up (or microcosting) approach, where units of health service are multiplied by their appropriate unit cost price and summed to provide an overall total cost estimate [11]. Costs will be measured in the local currency, but for the economic evaluation, they will be converted to international euro (Int.€) using purchasing power parity that takes into account exchange rates and the respective buying power in the countries. The reference year for the costs will be 2018.

### Data Handling

Data will be handled in accordance with the General Data Protection Regulation [12]. A central Study Protocol and Data Management Plan coordinates all of the five independent trials. In addition, each trial will have a data entry template, a locally adapted version of the study protocol, and a locally adapted version of the central project data safety and management plan.

### Sample Size Calculation

Each of the trials is well powered with 90 participants per randomization group (n=180 in total) to detect a clinically relevant effect (mean standardized difference, *d*) of ≥0.33 as statistically significant (at α≤.05, two-tailed) with a power (1-β) of ≥0.80 when the primary outcome (WHODAS personal and social functioning) is evaluated in a baseline-adjusted analysis of variance or similarly specified regression model.

More specifically, the power calculation is carried out with the sample size procedure of Stata (Stata Corp, College Station, Texas, USA) (*sampsi* [13]) assuming that the effect evaluation of functioning would be carried out in a baseline-adjusted analysis of covariance (ANCOVA) with one baseline measurement and two follow-ups. We had to make assumptions about the strength of the correlation of functioning between *t_0_* and *t_1_* and between *t_1_* and *t_2_* (denoted as *r_0,1_* and *r_1,2_*, respectively). The WHODAS 2.0 has a high 1-week test-retest reliability of *r_0,1_*=0.98 [14], but the correlation between *t_0_* and *t_1_* will be lower when the measurements are further apart (12-month time interval between *t_0_* and *t_1_*). Hence, we assumed that *r_0,1_* would be in the more modest range of 0.30 to 0.50. Regarding the strength of the correlation of personal and social functioning between *t_1_* and *t_2_*, we made a similar assumption, but expect that this correlation will be weaker still (ie, in the range of 0.20 to 0.40). The size of these correlations is important because they affect the required sample size. For this reason, the sample size calculations were repeated for the likely range of *r_0,1_* and *r_1,2_*. Table 1 shows the required sample size (per arm) for varying *r_0,1_* and *r_1,2_* values.

**Table 1:** Required sample size per condition for varying *r_0,1_* and *r_1,2_* values.

*r_1,2_* ^a^	*r_0,1_* ^b^		
	0.3	0.4	0.5
0.2	74	64	51
0.3	81	71	58
0.4	88	78	65

^a^Correlation of functioning between 12 months after baseline (*t_1_*) and 18 months after baseline (*t_2_*).

^b^Correlation of functioning between baseline (*t_0_*) and 12 months after baseline (*t_1_*).

Table 1 shows that in all possible scenarios, the study would be well powered with 88 participants (say 90 participants per condition or 180 in total). In fact, there is a chance that a smaller participant number would suffice, but it is better to be safe. As indicated, the power is based on the idea that the analyses will be conducted with ANCOVA repeated measures or an equivalently specified linear mixed regression model. It was therefore tested if a total sample size of 180 is sufficient for detecting the effect of *d*≥0.33 as statistically significant at α≤.05 (two-tailed) with a power of 0.80, when mixed modelling is used. Hence, to determine the sample size required to achieve 80% power, 1000 mixed model simulations were performed, in which baseline measurement and a random effect for individuals were considered. In generating the simulation data, correlations of 0.3 and 0.1 (Pearson *r*) were assumed between *t_0_* and *t_1_* and between *t_1_* and *t_2_*, respectively. Furthermore, a treatment effect of 0.33 was assumed. In doing so, a sample size of 180 was found to be sufficient.

We will not compensate for dropout by increasing the number of participants at baseline, unless the local research teams identify additional opportunities to recruit more patients than the minimum of 180. As part of the ITT analysis, all participants will be analyzed as randomized, and this will be achieved by either using mixed modelling or imputing missing observations. Imputation is not only required to persevere randomization integrity, but will in addition restore power losses due to dropout. In summary, local teams will have to recruit 180 participants, but can recruit more (up to a maximum of 200) to compensate for dropout when logistically feasible.

It should be noted that the power analysis is directed at the evaluation of the central clinical end-term functioning (ie, the alternative hypothesis predicts that functioning will be at least 0.33 standard units better in the CMHC condition compared with the CAU condition). It is not customary to power a study for testing a health-economic hypothesis, because the large standard errors associated with costs would require extremely large sample sizes. Instead, in health-economic evaluation, a probabilistic medical decision-making approach is used for making inferences about the relative cost-effectiveness of CMHC compared with CAU.

Finally, it is worth mentioning that both the clinical and health-economic evaluations will be based on the pooled dataset of all 900 participants when the data of all five trials are combined.

### Analysis

The health-economic evaluation will be conducted as a CEA with health care costs (in euro for the reference year 2018) related to WHODAS treatment response (WHODAS functioning dichotomized) and a CUA of incremental costs per QALY gained. These analyses will be conducted from the health care system perspective and hence will have a focus on health care costs and health-related outcomes. Sensitivity analyses will be carried out and will be directed at the main cost drivers and uncertainty in the outcomes. Sensitivity analyses are conducted to assess the robustness of the main analyses of the CEA and CUA or to enrich the analyses by repeating them in a different way. In one of the planned sensitivity analyses, the CEA and CUA are expanding the health care system’s perspective to a wider societal perspective (with changes in productivity included). Finally, the statistical analyses will be based on the pooled dataset of all five trials. Owing to the lack of country-specific guidelines for each of the participating sites, the guidelines of the UK National Institution for Care Excellence will be used [15,16].

### Costing

Total costs will be estimated using a bottom-up (or microcosting) approach, where units of health service are multiplied by their appropriate unit cost price and summed to provide an overall total cost estimate [11]. Unit costs will be determined for Croatia and will be extrapolated to the other participating countries based on the purchasing power parity of the respective countries. Cost prices will be estimated using microcosting based on hospital records, financial departments, and national tariffs. Microcosting takes into consideration (if applicable) the initial investment for equipment, other investments, maintenance, number of years of use and discounting, material costs, personnel costs (per hour), and an increase for the overhead of the respective unit price. Costs of medication (and dispensing costs) will be calculated using daily defined dosage (based on clinical practice guidelines) and data from the financial departments of the five participating hospitals, indicating the mean medication usage per adult a day. Productivity losses will only be included in the analyses in which a societal perspective is adopted. Productivity losses will be evaluated using both the friction cost approach (ie, calculation of productivity losses solely for a prespecified “friction period” in which an employee would have been replaced) and the human capital approach (ie, calculation of the productivity losses for the full period of absenteeism) [17,18]. Furthermore, costs of informal care will be based on the shadow prices for unpaid work in the respective countries. Costs of transport will be calculated as the mean distance per destination to the health care provider multiplied by tariffs of public transport. Total costs will be aggregated over time by calculating the area under the curve (AUC). All costs will be expressed in euro for 2018. If necessary, existing cost prices will be updated to 2018 values using the consumer price index.

All costs will be converted to Int.€ using purchasing power parity, which makes it possible to compare costs between countries with different standards of living (to derive a uniform currency by equalizing the purchasing power of different currencies through the elimination of differences in price levels between countries [19]). Following the National Institute for Care Excellence (NICE) guidelines for health-economic evaluation, both costs and effects will be discounted by 3.5% per annum, because the time horizon of the trials extends beyond 1 year. However, discounting rates will be subject to sensitivity analysis because discounting can have a substantial impact on the outcomes of a health-economic evaluation.

### Cost-Effectiveness Analysis

The CEA, with costs and WHODAS functioning as the primary outcomes, will be conducted in several steps.

First, WHODAS global functional disability will be computed using the scoring algorithm based on the item response theory as recommended by WHO [6]. This algorithm provides a per patient functional disability score between 0 and 100, with higher scores indicating greater disability.

Second, the sample characteristics at *t_0_* will be described to see if despite randomization, some baseline imbalances across conditions have occurred in prognostically relevant variables (ie, variables that are strongly correlated with the outcome). If this is the case, such variables will be used as covariates to make adjustments for the baseline imbalances. In the unlikely scenario where many potential confounders are found, covariate adjustments will be made more efficiently using inverse propensity score weighting, with w=1/p in the control group and w=1/(1-p) in the experimental group, where *w* is the weight and *p* is the propensity score (ie, the likelihood that a participant is in one condition rather than the other), and *p* will be estimated under a logistic model.

Third, dropout will be evaluated, and missing observations in WHODAS functioning at *t_1_* and *t_2_* will be imputed to permit ITT analysis. The imputation will be based on the predictors of outcome (for accuracy) and predictors of missing values (to adjust for possibly selective dropout). To handle missing data, single imputation using predictive mean matching embedded in nonparametric bootstraps of seemingly unrelated regression equations (SURE model) will be used. In a recent paper by Brand et al, single imputation nested in the bootstrap percentile method emerged as the method with the best statistical properties [20]. Predictive mean matching will be used to account for nonnormality of the data by imputing “real” observed values from similar cases instead of imputing regression estimates [21,22].

Fourth, in the context of the economic evaluation, WHODAS global functioning must be dichotomized into a binary treatment response outcome. This is done because from an economics perspective, it is meaningless to relate hard currency (euro, which is measured at the interval level) to a health gain (measured at an “elastic” ordinal measurement level). In other words, for a health-economic evaluation, a “tangible” outcome on par with the hard currency required to generate that health outcome is needed in order to merit a meaningful analysis. In this analysis, a binary *treatment response* variable (1, improved; 0, not improved) would constitute such a hard outcome. For the main analysis, treatment response is defined when a patient has improved by 0.33 standard units or more. A change of 0.33 standard units is equivalent to a 6-point change on the WHODAS 0-100 scale. Thus, when a patient has improved 6 points or more, the patient is considered as a treatment responder (ie, treatment response=1 or else 0).

Fifth, to simultaneously evaluate both costs and outcomes, SURE models will be used. The SURE models will be baseline adjusted with baseline WHODAS functioning and cost as covariates or weighted with inverse propensity scores as needed. Because costs are nonnormally distributed, the SURE models will be bootstrapped (2500 times). When bootstrapping, one creates N times (in this case 2500 times) a new sample out of the original sample with replacement. This results in N different samples. Incremental cost-effectiveness ratios (ICERs) will be computed by dividing the between-condition differences in costs by the difference in effects (treatment responders). Thereafter, the scatter of 2500 bootstrapped ICERs will be plotted on the ICER plane. When most simulated ICERs fall into the north-east quadrant of the ICER plane (indicating that better health is achieved at higher costs), an acceptability curve will be graphed for decision-making purposes. The acceptability curve depicts the likelihood that the new health care system has acceptable cost-effectiveness relative to CAU given varying willingness-to-pay ceilings for gaining a QALY [23].

These health-economic evaluations will answer the question, “To what extent the newly implemented community-based recovery-oriented health care system has better patient-level outcomes with regard to WHODAS functioning?”

### Cost-Utility Analysis

The methods for the CUA are the same as those for the CEA, with the exception that the incremental costs per QALY gained is the primary outcome. The QALYs will be computed from the EQ-5D-3L and will be based on the Slovenian VAS-based tariffs and the AUC method [9]. The Slovenian tariffs will be used in the absence of local tariffs for the participating countries, and hence, the Slovenian tariffs are deemed most representative. In the sensitivity analyses, alternative strategies for computing the QALYs will be used.

### Sensitivity Analyses

The analyses mentioned above will be subject to a series of sensitivity analyses to gauge the robustness of the main findings. Sensitivity analyses will be directed at several uncertainties. First, the health-economic evaluation in the main analysis is restricted to the health care system’s perspective, where the costs are confined to the health care costs incurred by mental health services. In the sensitivity analysis, the health care perspective will be broadened to include the out-of-pocket costs of the patients and their family members for informal copayments, traveling costs for trips to healthcare centers, and informal care. In addition, the costs and benefits stemming from changes in productivity losses will be included. These costs stem from sickness absence (absenteeism) and lesser efficiency while at work (presenteeism). Second, in the main analysis, the valuation of the EQ-5D health states (ie, the tariffs) will be based on the Slovenian tariffs. For the sensitivity analysis, the tariffs will also be based on the study by Greiner et al, which is representative of West European countries, but might be less valid for Central and East European countries [24]. In addition, for each of the participating countries, we will repeat the main analysis using the country-specific EQ-5D VAS. Third, extreme cost outliers in the data may exert a disproportional influence on the economic evaluation. In the sensitivity analysis, we will rerun the economic evaluation while winsorizing cost data (ie, replacing the top 10% highest costs by more modest costs corresponding with the 90th percentile) [25]. Fourth, the choice of the discounting rates may impact the outcomes of the health-economic evaluation and will therefore be varied between 1% and 5% for both the costs and QALY gains. In a sensitivity analysis, the main analyses will be repeated with an annual discounting rate of 3.5% for the effects and 4.0% for the costs, as per the Dutch guidelines for health-economic evaluation [26].

The sensitivity analyses will help to assess the robustness of the findings that were obtained under the main analysis and will enrich the main analysis by taking different perspectives.

### Analysis of Pooled Trial Data

One of the secondary goals of the RECOVER-E project is to support and develop on-site research skills and to strengthen collaboration between countries. Therefore, the health-economic evaluations will be carried out locally at each of the sites. Central analysis will also be conducted for the pooled dataset of 900 (5 × 180) participants. The pooled data will be analyzed using mixed linear models with random effects both at the patient and site levels (equivalent to individual participant data meta-analysis) or alternatively with design-based analysis for the data of participants clustered at sites. The pooled data analysis, which has greater statistical power to detect significant effects, will include WHODAS functioning (on the continuous scale), as well as treatment response (dichotomized) and EQ-5D QALY gains. Finally, the pooled data will allow for multilevel modelling of net monetary benefits as the outcome of interest, with net benefits defined as NB = E*λ- C, where NB represents the net benefits, E represents the effects, λ represents a varying willingness-to-pay value (in euro) for gaining one unit of E, and C represents the costs required for generating that one unit health gain.

### Reporting

The above evaluations will be reported in agreement with the following pertinent guidelines: the CONSORT statement for randomized trials [27], Consolidated Health Economic Evaluation Reporting Standards (CHEERS) statement for trial-based health-economic evaluation [28], and Consolidated Framework for Advancing Implementation Science [29].

## Results

Data collection was started in December 2018 (Croatia), February 2019 (Montenegro), April 2019 (Romania), June 2019 (North Macedonia), and October 2019 (Bulgaria). At the time of acceptance of this manuscript, the following numbers of participants were included at each site: 91 in Bulgaria, 165 in Croatia, 180 in Romania, 197 in Montenegro, and 190 in North Macedonia. All procedures are in accordance with the ethical standards of the ethics committees of the participating countries and with the 1964 Helsinki Declaration and its later amendments or comparable ethical standards. Informed consent will be obtained from all individual participants included in the study. The five trials have been registered separately for every site. The registration numbers on ClinicalTrials.gov are as follows: NCT03922425 (Bulgaria), NCT03862209 (Croatia), NCT03892473 (Macedonia), NCT03837340 (Montenegro), and NCT03884933 (Romania). The results from the various evaluations will be summarized in policy briefs (a part of the policy influencing strategies developed in each country) using clear and nontechnical wording. The policy briefs will inform decision-makers about the project findings during the final policy dialogue sessions (one per site). Papers reporting primary outcomes will be published in open-access journals and findings will be presented in other academic and scientific fora as per the RECOVER-E research dissemination strategy [30]. The first results describing the follow-up data are expected in 2021.

## Discussion

### General Considerations

This study will examine the cost-effectiveness of recovery-oriented community mental health care for patients with severe mental disorders (the intervention implemented in the RECOVER-E project) compared with CAU in Bulgaria, Croatia, Macedonia, Montenegro, and Romania. Health-economic evaluations will be conducted alongside hybrid effectiveness-implementation trials at each of the five sites. In addition, a pooled analysis will be performed combining all trial data. It is hypothesized that the shift toward deinstitutionalization using a locally adapted form of flexible assertive community treatment results in the reduction of health care costs by avoiding expensive emergency care or psychiatric hospitalization. At the same time, this intervention has a focus within service delivery on recovery goals, which is hypothesized to contribute to a greater sense of societal role fulfilment and participation in society among people with severe mental illness. It is not unlikely that patients receiving community care will show larger improvements in WHODAS personal and social functioning and EQ-5D health-related quality of life as compared with patients treated in hospital-based mental health care services.

Given the nature of the intervention, a pragmatic approach is chosen to implement and evaluate community mental health services. While this may affect internal validity (eg, due to the lack of allocation concealment and masking), the corresponding results may be more generalizable and applicable to routine practice settings [31]. Moreover, the proposed methodology includes some methodological solutions to combat threats to the internal validity of the trials (eg, inverse propensity score weighting when randomization appears to be suboptimal).

There are additional limitations that are anticipated and worth noting. Among these, the lack of country-specific EQ-5D tariffs and the difficulty to obtain unit cost prices in lieu of national standard cost prices are relatively important. In an effort to address this lack of tariffs and standard cost prices, the EQ-5D VAS will be used to obtain QALYs and the microcosting technique will be used to obtain reasonably accurate local cost prices. Nonetheless, these approaches may introduce bias in the QALY and cost estimates. It is hoped that these biases occur to the same degree in the CMHC condition as in the CAU condition of the trials and will therefore cancel each other out when computing cost differences and effect differences across the conditions. Moreover, robust statistical techniques will be used, such as nonparametric bootstrapping and inverse propensity score matching. Furthermore, the main analysis will be subject to various sensitivity analyses precisely directed at uncertainties in costs and outcomes in order to ascertain the robustness of the main analysis.

The use of the WHODAS self-report version instead of the interviewer-administered version may cause some reporting bias (eg, due to differences in the literacy level between participants). However, given the randomized nature of the study, we expect this bias to be present equally in both arms. Furthermore, the self-reported version of the WHODAS has been demonstrated to identify improvements in functioning following treatment in people who have certain health conditions (eg, depression, schizophrenia, and alcohol problems) [7]. Regarding the use of the EQ-5D-3L, there is some evidence demonstrating a lack of responsiveness in patients with schizophrenia [32]. However, the EQ-5D-3L is recommended as the preferred utility instrument in most countries worldwide, and in line with the recommendations of Payakachat et al [32], we believe that an appropriate estimate of effectiveness is ensured by also using the WHODAS.

Although the operationalization of the societal perspective is challenging, in this study, we believe the use of the term “societal” is justified, as we include relevant societal costs, such as informal care, travel distances to health services, and productivity losses stemming from functional impairment. However, the educational and criminal justice sectors, which are often overlooked, may also be considered [33]. In this study, we feel that these sectors are relevant to a lesser extent and the substantial efforts in collecting data within these sectors is not justified by the expected impact on total costs (especially for the educational sector as we include adults only).

Lastly, we expect that it will be difficult for some of the participating study sites to recruit 180 participants into the trials and that the trials are likely to experience loss to follow-up. This may deflate the sample size and power. In the event of this occurring, power-efficient statistical techniques will be employed, such as baseline-adjusted ANCOVA (repeated measures) and similarly specified linear mixed models for ITT analysis. It is also worth noting that the economic evaluation will be conducted on the pooled dataset of all five trials combined, which will mitigate power issues, if any.

### Conclusions

All five countries included in this project are either relatively new EU members (from 2007 onwards) or EU candidates with per capita GDP far below the EU average. Consequently, their health care budgets are constrained and also face many competing priorities. In this context, scientifically sound health-economic evaluation is a prerequisite for policy makers to decide on wider, possibly national, implementation and scale up of community-based recovery-oriented mental health services. In addition, the results of the health-economic evaluation will contribute to the growing evidence base of effective and cost-effective recovery-oriented and community-based service models for sustainable mental health systems for people with severe and enduring mental ill health in low- and middle-income countries.

## Appendix

Multimedia Appendix 1 CONSORT 2010 flow diagram for the RECOVER-E program.

## Abbreviations

ACT: assertive community treatment
ANCOVA: analysis of covariance
AUC: area under the curve
CAU: care as usual
CEA: cost-effectiveness analysis
CHEERS: Consolidated Health Economic Evaluation Reporting Standards
CMHC: community-based recovery-oriented mental health care
CMHT: community mental health care team
CONSORT: Consolidated Standards of Reporting Trials
CUA: cost-utility analysis
EQ-5D: EuroQol five dimensions
EQ-5D-3L: three-level EuroQol five dimensions
FACT: flexible assertive community treatment
ICER: incremental cost-effectiveness ratio
ITT: intention-to-treat
QALY: quality-adjusted life year
SURE: seemingly unrelated regression equations
TiC-P: Trimbos/iMTA Questionnaire on Costs associated with Psychiatric illness
VAS: visual analog scale
WHO: World Health Organization
WHODAS 2.0: World Health Organization’s Disability Assessment Schedule 2.0
