# Supplementary material for: Sustainability of Community-Based Specialized Mental Health Services in Five European Countries: Protocol for Five Randomized Controlled Trial–Based Health-Economic Evaluations Embedded in the RECOVER-E Program
Source: JMIR Res Protoc. 2020 Jun 1;9(6):e17454. doi: 10.2196/17454 (PMC7296406; doi:10.2196/17454)
Supplement: Multimedia Appendix 1 [file resprot_v9i6e17454_app1.doc]

**
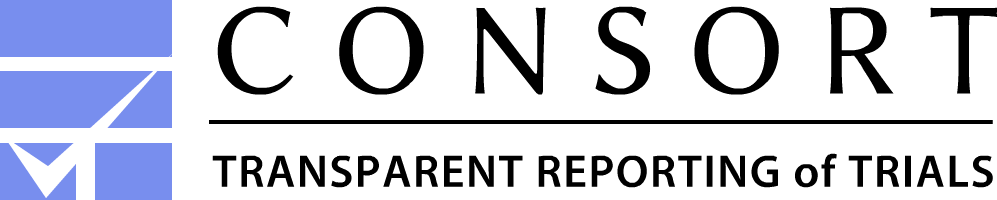
CONSORT 2010 Flow Diagram**

Assessed for eligibility (n= unknown)

(Consenting adults (aged 18-65) with severe mental illness, defined as:

- severe depression, bipolar disorder, or schizophrenia as per the DSM-5 or ICD-10, not in symptomatic remission
- severe limitations in personal and social role functioning (as per the International Classification of Functioning, Disability and Health, ICF), not in functional remission, hence indicated for coordinated care provided by multidisciplinary community mental healthcare teams

)

**Analysis**

**Follow-Up**

**Enrollment**

**Allocation**

Excluded (n= unknown)

♦ somatic comorbidities that require prolonged medical care in a hospital,

♦ incarcerated patients

♦ terminally ill patients

Analysed (n= unknown)

For each site:
♦ Intention to treat protocol
♦ Multi-level analyses to determine (clinical) effectiveness
♦ Economic evaluation from a healthcare perspective (non-parametric bootstrapping)
♦ Excluded from analysis (n= unknown)

Pooled analysis for all five countries together

**Measurements at baseline and after 12 and 18 months**♦ Lost to follow-up (n= unknown) ♦ Discontinued intervention (n= unknown)

**Measurements at baseline and after 12 and 18 months**♦ Lost to follow-up (n= unknown) ♦ Discontinued intervention (n= unknown)

Randomized (n= 180)

Allocated to CMHT (n= 90)

♦ Received allocated treatment (n= unknown)

♦ Did not receive allocated treatment (n= unknown)

Allocated to care as usual (n= 90)

♦ Received allocated treatment (n= unknown)

♦ Did not receive allocated treatment (n= unknown)
